# Anti-Factor XII Autoantibodies in Patients with Recurrent Pregnancy Loss Recognize the Second Epidermal Growth Factor–Like Domain

**DOI:** 10.1055/s-0039-1695709

**Published:** 2019-09-03

**Authors:** Yoshihiro Sato, Toshitaka Sugi, Rie Sakai

**Affiliations:** 1 Laboratory for Recurrent Pregnancy Loss, Sugi Women's Clinic, Yokohama, Japan; 2 Yoshihiro Women's Clinic, Tokyo, Japan

**Keywords:** factor XII, pregnancy loss, recurrent pregnancy loss, epidermal growth factor, heparin-binding EGF-like growth factor

## Abstract

**Background** Factor XII (FXII) deficiency and autoantibodies that bind to FXII (anti-FXII) have been described in patients with adverse pregnancy outcomes, including recurrent pregnancy loss. It has been reported that FXII functions not only as a coagulation protein but also as a growth factor.

**Objectives** We studied the association between anti-FXII and the epidermal growth factor (EGF) system in patients with recurrent pregnancy loss.

**Patients/Methods** We used synthetic peptides that span the second EGF-like domain in the heavy chain of FXII (EGF2) in inhibition and direct binding studies to determine if anti-FXII antibodies recognize EGF2. Furthermore, we examined whether anti-FXII antibodies, which recognize EGF2, also recognize recombinant EGF and heparin-binding EGF-like growth factor (HB-EGF).

**Results** Among 100 patients with recurrent pregnancy loss, the plasma of 23 patients (23.0%) recognized the synthetic peptide ASQ41, which covers EGF2. Among the 23 anti-ASQ41-positive patients, plasma samples from 13 patients (56.5%) recognized the 22-residue segment C-terminal half of ASQ41. Among the 23 anti-ASQ41-positive patients, the plasma of 17 patients (73.9%) recognized recombinant human EGF. Affinity-purified anti-FXII antibodies, which recognize ASQ41, also recognized recombinant EGF family proteins such as EGF and HB-EGF.

**Conclusions** The autoantibodies in patients with recurrent pregnancy loss recognized the EGF2 domain in FXII and other proteins of the EGF family. Since proteins in the EGF family play an important role in normal pregnancy, autoantibody-associated disruption of the EGF system may cause pregnancy loss.

## Introduction

Recently, several studies have observed associations between recurrent pregnancy loss and factor XII (FXII) deficiency. 1 2 3 Schved et al 1 reported cases where three young women with FXII deficiencies had a clinical history of spontaneous abortion. Braulke et al 2 reported eight patients with moderately reduced levels of FXII among 43 patients with recurrent pregnancy loss. Gris et al 3 evaluated the prevalence of hemostatic abnormalities in 500 consecutive women with unexplained recurrent pregnancy loss. They found that 9.4% of patients exhibited an isolated FXII deficiency.

Some reports have revealed a high incidence (20.9%) of FXII deficiency in patients who were positive for lupus anticoagulant (LA). 4 They hypothesized that anti-FXII autoantibodies (anti-FXII) might be present in several patients who were positive for LA and that the possible formation of immune complexes led to reduced FXII levels. They reported that many LA-positive patients were also positive for anti-FXII according to enzyme-linked immunosorbent assay (ELISA) and surface plasmon resonance. 5

Although some studies have reported that FXII deficiency is a risk factor for recurrent pregnancy loss, 1 2 3 others failed to find such an association. 6 Pauer et al generated mice with FXII deficiency using a gene-targeting approach. 7 Interestingly, they reported that normal litter sizes resulted from mating FXII ^−/−^ male mice and FXII ^−/−^ female mice, which suggests that total FXII deficiency did not affect pregnancy outcomes. 7 Iwaki and Castellino also revealed that in female mice homozygous for FXII deficiency, normal deliveries with normal litter sizes were observed. 8

Jones et al 9 revealed that, compared with the levels of FXII in patients without anti-FXII antibodies, the levels of FXII were significantly lower in patients with anti-FXII. This suggests that reduced levels of FXII resulted from the formation of immune complexes and subsequent sequestration. They also suggest that the presence of anti-FXII demonstrated a strong and significant correlation with recurrent pregnancy loss. 10 Autoantibodies to FXII, rather than FXII deficiency, may therefore be a risk factor for recurrent pregnancy loss.

Epitope mapping analysis indicated that antibodies from most patients with recurrent pregnancy loss recognized the heavy chain of FXII, but not the light chain, and that the antigen-binding site of anti-FXII comprises amino acids 1–30 (IPP30) in the heavy chain of FXII. 11 Among plasma samples from 17 patients with recurrent pregnancy loss who were positive for anti-FXII antibodies, the antibodies from 13 patients (76.5%) recognized the IPP30 peptide. Bradford et al revealed that activated FXII inhibited the interaction between thrombin and platelets. 12 They also revealed that binding of FXII to platelets was inhibited by a monoclonal antibody, whose epitopes have been mapped to amino acids 1–28 and amino acids 134–153 in the fibronectin type I domain of FXII. 13 14 This suggests that anti-FXII species that recognize IPP30 in patients with recurrent pregnancy loss may inhibit FXII binding to platelets, which leads to thrombosis and pregnancy loss. Results from our previous study 15 supported this hypothesis and demonstrated that exogenously added polyclonal antibodies against IPP30 markedly increased γ-thrombin-induced platelet aggregation in vitro. Furthermore, it was reported that, in a murine model, anti-FXII infusion (or infusion of anti-IPP30 antibodies) induced placental thrombosis and hemorrhage as well as increased placental apoptosis. 16 In addition, fewer mitotic cells, decreased trophoblast giant cell invasion, and more shrunken cells in the decidua were observed.

Most studies have focused on FXII as a coagulation protein that is essential for surface-activated blood coagulation tests. However, it has also been reported that FXII functions as a growth factor. 17 18 19 FXII consists of a fibronectin type II domain (Fib2), an epidermal growth factor (EGF)-like domain (EGF1), a fibronectin type I domain (Fib1), a second EGF-like domain (EGF2), a kringle domain, a proline-rich region, and a catalytic domain. In our previous epitope mapping study, the N-terminal half of the FXII heavy chain including EGF1 was examined; however, EGF2 has not yet been studied. 11

In the present study, we used synthesized peptides for inhibition and direct-binding studies to determine if anti-FXII species in patients with recurrent pregnancy loss recognize EGF2. Furthermore, we examined whether autoantibodies against EGF2 (anti-EGF2) in patients with recurrent pregnancy loss recognize other proteins of the EGF family.

## Materials and Methods

### Patients

From December 2017 to March 2018, plasma samples from 100 patients with recurrent pregnancy loss who were referred to Sugi Women's Clinic (Yokohama, Japan) were studied.

Patients with recurrent pregnancy loss had three or more pregnancy losses before 10 weeks of gestation. Pregnancy losses did not include ectopic pregnancies or elective abortions. Each patient was evaluated for uterine anomalies according to three-dimensional vaginal ultrasound, endocrine monitoring (e.g., hemoglobin A _1C_ , fasting blood sugar, and thyroid function tests), FXII activity, anti-FXII, antinuclear antibodies, anti-EGF2, and the presence of antiphospholipid antibodies such as LA, anticardiolipin (aCL) antibodies, and antiphosphatidylethanolamine (aPE) antibodies. The mean age of the patients was 35.8 (range: 24–48) years, and the mean number of pregnancy losses was 3.2 (range: 3–5). No patient had a history of thrombosis. In terms of autoimmune disease, three patients had Hashimoto's thyroiditis. When we obtained plasma specimens, no patient was pregnant or had received any treatment.

LA was measured by the dilute Russell's viper venom time (dRVVT) method using a dRVVT kit purchased from Medical & Biological Laboratories (Nagano, Japan) according to the manufacturer's recommendations.

The aCL were measured by ELISA using a MESACUP kit purchased from Medical & Biological Laboratories (Nagano, Japan) according to the manufacturer's recommendations.

The level of aPE was measured by ELISA following a previously described method. 17 18 19 We screened patients for aPE because our previous study suggested that 37.5% of patients with recurrent pregnancy loss who were positive for anti-FXII antibodies were also positive for aPE and that these autoantibodies may have similar structures and functions. 15

We also recruited 65 healthy female volunteers who were not pregnant and who had no history of miscarriage. Blood samples were collected in nonactivating plastic tubes including 0.109 mol/L trisodium citrate (9:1 v/v). After centrifugation, plasma aliquots were immediately stored at −60°C until use. We obtained informed consent from all participants, and the local ethics review committee approved the study protocol.

### Materials

We purchased FXII and activated FXII from Enzyme Research Laboratories (South Bend, Indiana, United States) and the polyclonal antibodies against recombinant human EGF (antihuman EGF) from Abcam (Tokyo, Japan). Polyclonal antibodies against recombinant mouse EGF (antimouse EGF) were purchased from R&D Systems Inc. (Minnesota, United States), and recombinant mouse EGF was obtained from ProSpec-Tany TechnoGene Ltd. (Ness Ziona, Israel). Recombinant human EGF was purchased from Higeta Shoyu Co., Ltd. (Ibaraki, Japan), and recombinant human heparin-binding EGF-like growth factor (HB-EGF) was obtained from PeproTech (New Jersey, United States).

### FXII Activity

FXII activity was measured by a one-step clotting method using FXII-deficient plasma, which we purchased from Instrumentation Laboratory (Massachusetts, United States), according to the manufacturer's recommendations.

### SDS-PAGE and Immunoblotting for Anti-FXII

We performed sodium dodecyl sulfatepolyacrylamide gel electrophoresis (SDS-PAGE) using 10% polyacrylamide gels. Activated FXII was prepared in its reduced form, in which FXII was divided into the heavy chain and light chain. To prepare reduced activated FXII, dithiothreitol (DTT) was added to activated FXII. After vortexing, we boiled the mixture at 100°C for 5 minutes. Activated FXII (5 µL of a 71.2 µg/mL solution) was applied to each lane. Subsequent transfer to polyvinylidene difluoride (PVDF) membranes was performed for 20 minutes at 0.1 amps. Membranes were then blocked for 2 hours with 1% bovine serum albumin (BSA) in Tris-buffered saline (TBS; 0.02 mol/L Tris, 0.15 mol/L NaCl, pH 7.3). After incubation with plasma derived from patients or normal controls (1:100) for 2 hours, we performed three washes in 0.05% Tween 20/TBS. The membranes were then exposed to horseradish-peroxidase-conjugated polyclonal antibodies to human immunoglobulin G (IgG) or immunoglobulin M (IgM) for 1 hour, followed by washing as described above. The immunoreactive bands were developed using 3,3′,5,5′-tetramethylbenzidine. In our immunoblot, we evaluated the intensity of immunobands visually compared with negative controls. The intensity of immunobands was classified into five groups, namely −, +, ++, ++ + , or ++ + +. A plasma was considered positive if the intensity of immunobands was grouped in +, ++, ++ + , or ++ + +.

### Synthesis of Peptides

**Fig. 1.**
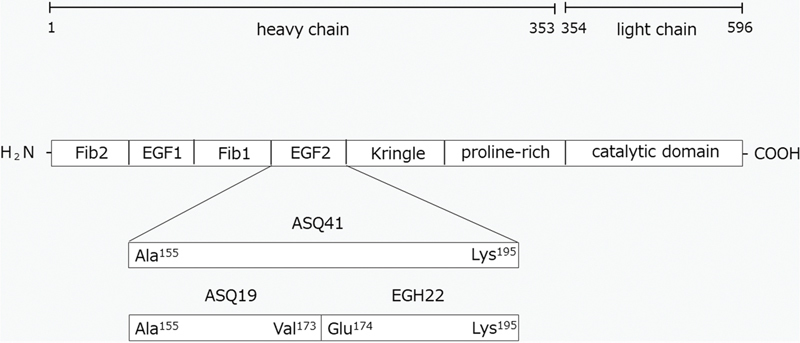
Domain structure of factor XII (FXII). FXII consists of a fibronectin type II domain (Fib2), an epidermal growth factor (EGF)-like domain (EGF1), a fibronectin type I domain (Fib1), a second EGF-like domain (EGF2), a kringle domain, a proline-rich region, and a catalytic domain. In the present study, ASQ41 (EGF2), ASQ19 (N-terminal half of EGF2), and EGH22 (C-terminal half of EGF2) were synthesized.

Peptides were synthesized (SCRUM Inc., Tokyo, Japan) as previously described. 11 16 ASQ41 (Ala ^155^ –Lys ^195^ of FXII; EGF2), ASQ19 (Ala ^155^ –Val ^173^ of FXII; N-terminal half of EGF2), EGH22 (Glu ^174^ –Lys ^195^ of FXII; C-terminal half of EGF2), and each scramble peptide (CRG41, CLR19, and HDT22) were synthesized ( Fig.1 and Table 1 ).

**Table 1:** Synthetic peptides used in the study

Peptides a	Sequence b	Positions
ASQ41	ASQACRTNPCLHGGRCLEVEGHRLCHCPVGYTGPFCDVDTK	155–195 of human FXII
CRG41	CRGEPHALALSREGCNVQTGCCVHTLCPHGCKGRDTFPVYD	Scramble peptide of ASQ41
ASQ19	ASQACRTNPCLHGGRCLEV	155–173 of human FXII
CLR19	CLREGAVCTNGPACSEHRL	Scramble peptide of ASQ19
EGH22	EGHRLCHCPVGYTGPFCDVDTK	174–195 of human FXII
HDT22	HDTDGCLVFEHGYDTPCVKCRG	Scramble peptide of EGH22

a Peptides are identified by their three N-terminal residues using a one-letter code, followed by the total number of residues constituting the peptide.

b A one-letter code for amino acid residues is used.

### Purification of Autoantibodies to the Synthetic Peptide ASQ41 from Plasma

An ASQ41–agarose affinity column was generated, and autoantibodies to the synthetic peptide ASQ41 were purified from plasma using an AminoLink Plus Immobilization Kit (Thermo Fisher Scientific, Massachusetts, United States) according to the manufacturer's protocol. Briefly, the ASQ41 peptide was coated on the agarose beads to generate an ASQ41–agarose affinity column, and then, we applied the plasma obtained from patients to the ASQ41–agarose affinity column and eluted autoantibodies to ASQ41 using a glycine/HCl buffer at pH 3.0. We used blood samples from two patients with recurrent pregnancy loss who had high-titers of autoantibodies to ASQ41 and whose sample volumes were sufficient to do further examination.

### Binding of Autoantibodies to the Synthetic Peptide ASQ41 by ELISA

Amino plates (Sumitomo Bakelite Co., Ltd., Tokyo, Japan) were treated with 100 μL of 2% glutaraldehyde in TBS for 2 hours at 25°C. We then washed the plates twice with distilled water. The plates were coated with several concentrations of ASQ41 or the scramble peptide CRG41 in TBS overnight at 4°C. After each well was blocked for 1 hour with 2% BSA in TBS, the plates were incubated with 50 μL of several dilutions of plasma derived from patients or normal controls (1:100) for 70 minutes. We added alkaline-phosphatase-conjugated polyclonal antibodies to human IgG or IgM (Abcam, Tokyo, Japan), followed by the substrate solution. We washed the plates three times with TBS containing 0.05% Tween 20 after incubation with the coated peptides, blocking, and incubations with serum and antibody conjugate. Finally, after development, the optical density (OD) was evaluated at 405 nm using a para-nitrophenyl phosphate substrate. Color development was stopped by the addition of 75 μL of 3N NaOH when the OD of a positive control reached 1.0.

We considered a plasma sample to be positive if the difference between the OD of the ASQ41 peptide and that of the scramble CRG41 peptide was greater than 0.3. This was based on the data of 65 control individuals. From the data, the number of multiples of the median (MoM) that was equivalent to 95% of the ELISA values of this control population was 2.79. The OD equivalent to 2.79 MoM was 0.251. To ensure that the value is not borderline, 0.251 was rounded to 0.3.

In this ELISA, the antibody titer of the eluate from the ASQ41-affinity column that contained autoantibodies to ASQ41 purified from anti-ASQ41-positive patient plasma was 1/27 of patient plasma.

### Inhibition of Autoantibodies Binding to ASQ41 by Peptides by Immunoblot Assay

SDS-PAGE was performed using a polyacrylamide gradient gel (5–20%). The synthetic peptide ASQ41 or the scramble peptide CRG41 (20 µM solution) was applied to each lane. Subsequent transfer to a PVDF membrane was performed for 20 minutes at 0.1 amps. We used 1% BSA in TBS (pH 7.3) to block the membrane for 1 hour. The synthetic peptide ASQ41 or the scramble peptide CRG41 (20 µM solution) and 1 mL of IgM anti-ASQ41-positive patient plasma or normal control plasma diluted 1:100 in 1% BSA/TBS containing 0.03% Tween 20 were incubated with the membrane for 2 hours, followed by three washes in 0.05% Tween 20/TBS. Inhibition was achieved by incubating the liquid phase peptide and plasma with the solid-phase peptide coated on the membrane. The membranes were exposed to horseradish-peroxidase-conjugated polyclonal antibodies against human IgM for 1 hour, followed by washing as described above. The immunoreactive bands were developed using 3,3′,5,5′-tetramethylbenzidine.

### Polyclonal Antibodies against EGF Recognize FXII

FXII or activated FXII was subjected to SDS-PAGE under nonreducing or reducing conditions. The slab gel contents were transferred to PVDF membranes that were then immunoblotted with antimouse EGF or antihuman EGF.

### Statistical Analysis

Differences between the two groups were analyzed for statistical significance ( *p* < 0.05) by the Fisher's exact test.

## Results

### Binding of Autoantibodies to the Synthetic Peptide ASQ41 by ELISA

**Fig. 2.**
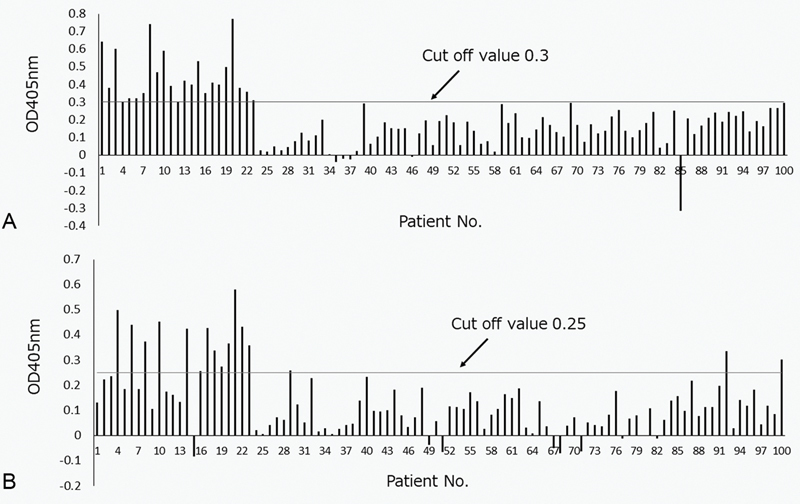
( **A** ) Binding of autoantibodies to the synthetic peptide ASQ41 by ELISA. ( **B** ) Binding of autoantibodies to the synthetic peptide EGH22 by ELISA. ELISA was performed using amino plates coated with 10 µM of the peptides or scramble peptides. IgM anti-ASQ41-positive patient plasma or normal control plasma (1:100) was applied, followed by an alkaline-phosphatase-conjugated secondary antibody. The absorption at 405 nm was then measured. ELISA, enzyme-linked immunosorbent assay; IgM, immunoglobulin M.

Among 100 patients with recurrent pregnancy loss, plasma samples from 23 patients (23.0%) recognized the ASQ41 peptide ( Fig. 2A ). These 23 patients were positive for IgM. No patient was positive for IgG. Therefore, only IgM antibodies were measured in subsequent experiments.

We found that 6, 0, 4, 2, and 3 of 100 patients were positive for LA, IgG aPE, IgM aPE, IgG aCL, and IgM aCL, respectively. As shown in Table 2 , a significant association was observed between IgM anti-ASQ41 and IgM aPE.

**Table 2:** Antiphospholipid antibodies, FXII deficiency, and anti-FXII in 100 patients with recurrent pregnancy loss

IgM anti-ASQ41	Positive	Negative	*p* -Value
Patients ( *n* )	23	77	
LA ( *n* , %)	0 (0.0)	6 (7.8)	NS
IgG aPE ( *n* , %)	0 (0.0)	0 (0.0)	NS
IgM aPE ( *n* , %)	3 (13.0)	1 (1.3)	0.0370
IgG aCL ( *n* , %)	1 (4.3)	1 (1.3)	NS
IgM aCL ( *n* , %)	1 (4.3)	2 (2.6)	NS
FXII deficiency ( *n* , %)	8 (34.8)	16 (20.8)	NS
IgM anti-FXII ( *n* , %)	8 (34.8)	8 (10.4)	0.0093

Abbreviations: aCL, anticardiolipin antibodies; anti-ASQ41, autoantibodies against the synthetic peptide ASQ41 that span the second epidermal growth factor-like domain in the heavy chain of factor XII; anti-FXII, autoantibodies against factor XII; aPE, antiphosphatidylethanolamine antibodies; FXII, factor XII; IgG, immunoglobulin G; IgM, immunoglobulin M; LA, lupus anticoagulant.

Among 23 anti-ASQ41-positive patients and 77 anti-ASQ41-negative patients, the incidences of FXII deficiency (<60%) and IgM anti-FXII were as follows: FXII deficiency, 34.8 versus 20.8% (NS); and IgM anti-FXII, 34.8 versus 10.4% ( *p* = 0.0093), respectively ( Table 2 ).

The incidence of IgM anti-FXII was significantly higher in IgM anti-ASQ41-positive patients than in anti-ASQ41-negative patients.

Among 100 patients with recurrent pregnancy loss, 16 patients (16.0%) were positive for IgM anti-FXII. Among 16 IgM anti-FXII-positive patients, plasma samples from eight patients (50.0%) recognized the ASQ41 peptide.

### Binding of Autoantibodies to the Synthetic Peptide EGH22 and ASQ19 by ELISA

We considered a plasma sample to be positive if the difference between the OD of the EGH22 peptide and the OD of the scramble HDT22 peptide was greater than 0.25. This result was based on the data of 77 anti-ASQ41-negative patients for which direct binding to synthetic peptides was tested by ELISA. On the basis of the data, the number of MoM that was equivalent to 95% of the ELISA values of this control population was 3.76. The OD equivalent to 3.76 MoM was 0.233. To ensure that the value is not borderline, 0.233 was rounded to 0.25.

We considered a plasma sample to be positive if the difference between the OD of the ASQ19 peptide and the OD of the scramble peptide CLR19 was greater than 0.2. This result was based on the data of 77 anti-ASQ41-negative patients for which direct binding to synthetic peptides was tested by ELISA. On the basis of the data, the number of MoM that was equivalent to 95% of the ELISA values of this control population was 2.43. The OD equivalent to 2.43 MoM was 0.112. To ensure that the value is not borderline, 0.112 was rounded to 0.2.

Among 23 patients with recurrent pregnancy loss who recognized the ASQ41 peptide, plasma samples from 13 patients (56.5%) recognized the EGH22 peptide ( Fig. 2B ). No patient plasma sample recognized the ASQ19 peptide.

### Dose-Dependent Binding of Autoantibodies to the Synthetic Peptide ASQ41

**Fig. 3.**
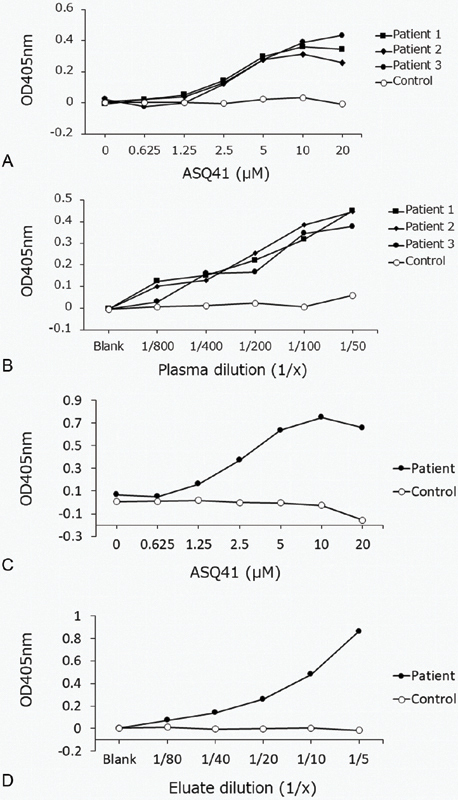
Dose-dependent binding of autoantibodies to the synthetic peptide ASQ41. ( **A** ) Amino plate wells were coated with increasing concentrations (35 µL of 0, 0.625, 1.25, 2.5, 5.0, 10, and 20 μM solutions) of ASQ41 or the scramble peptide CRG41. We then applied anti-ASQ41-positive patient plasma samples or normal control plasma (1:100), followed by an alkaline-phosphatase-conjugated secondary antibody. The absorption was evaluated at 405 nm. Then, we subtracted the OD value of the scramble peptide CRG41 from the OD value of the ASQ41 peptide. ( **B** ) Amino plate wells were coated with ASQ41 or the scramble peptide CRG41 (30 μL of a 10 μM solution). We then applied several dilutions (1:800, 1:400, 1:200, 1:100, and 1:50) of anti-ASQ41-positive patient plasma samples or normal control plasma, followed by an alkaline-phosphatase-conjugated secondary antibody. The absorption was evaluated at 405 nm. Then, we subtracted the OD value of the scramble peptide CRG41 from the OD value of the ASQ41 peptide. ( **C** ) Amino plate wells were coated with increasing concentrations (50 µL of 0, 0.625, 1.25, 2.5, 5.0, 10, and 20 μM solutions) of ASQ41 or the scramble peptide CRG41. We then applied the eluate from the ASQ41-affinity column that contained autoantibodies to ASQ41 that were purified from anti-ASQ41-positive patient plasma or normal control plasma. We then applied an alkaline-phosphatase-conjugated secondary antibody. The absorption was evaluated at 405 nm. Then, we subtracted the OD value of the scramble peptide CRG41 from the OD value of the ASQ41 peptide. ( **D** ) Amino plate wells were coated with ASQ41 or the scramble peptide CRG41 (50 μL of a 10 μM solution). We then applied several dilutions (1:80, 1:40, 1:20, 1:10, and 1:5) of the eluate, which contained purified antibodies to ASQ41 from anti-ASQ41-positive patient plasma or normal control plasma, followed by an alkaline-phosphatase-conjugated secondary antibody. The absorption was evaluated at 405 nm. Then, we subtracted the OD value of the scramble peptide CRG41 from the OD value of the ASQ41 peptide. OD, optical density.

Fig. 3 shows the binding of autoantibodies to ASQ41 in a concentration-dependent manner.

### Inhibition of Autoantibodies Binding to ASQ41 by Peptides

**Fig. 4.**
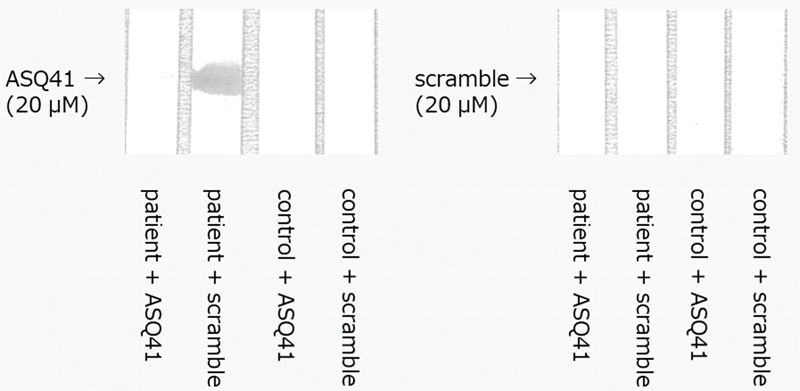
Inhibition of autoantibodies binding to ASQ41 by peptides. The synthetic peptide ASQ41 or the scramble peptide CRG41 (20 µM) was applied to each lane. After transfer to PVDF membranes, the membranes were incubated with the synthetic peptide ASQ41 or the scramble peptide CRG41 (20 µM solution) and either anti-ASQ41-positive patient plasma samples or normal control plasma. Next, immunoblotting was performed. PVDF, polyvinylidene difluoride.

As shown in Fig. 4 , ASQ41 blocked autoantibodies binding to ASQ41. This indicates that autoantibodies recognized not only solid-phase but also liquid-phase ASQ41.

### Binding of Autoantibodies to the Synthetic Peptide ASQ41 to Recombinant Human EGF

ELISA was performed using amino plates coated with 10 µM recombinant human EGF. Anti-ASQ41-positive patient plasma or normal control plasma (1:100) was applied. We considered a plasma sample to be positive if the difference between the OD of recombinant human EGF and the OD of TBS was greater than 0.22. This was based on the data of 65 normal individuals for which direct binding to recombinant human EGF was tested by ELISA. On the basis of the data, the number of MoM that was equivalent to 95% of the ELISA values of this control population was 2.44. The OD equivalent to 2.44 MoM was 0.22.

Among 23 anti-ASQ41-positive patients and 77 anti-ASQ41-negative patients, the incidences of antirecombinant human EGF antibodies were as follows: 73.9% (17/23) versus 32.5% (25/77) ( *p* = 0.0006), respectively.

Among 64 control individuals, the incidence of antirecombinant human EGF antibodies was 4.7% (3/64).

### Binding of ASQ41-Bound Autoantibodies Purified from Patient Plasma to FXII

**Fig. 5.**
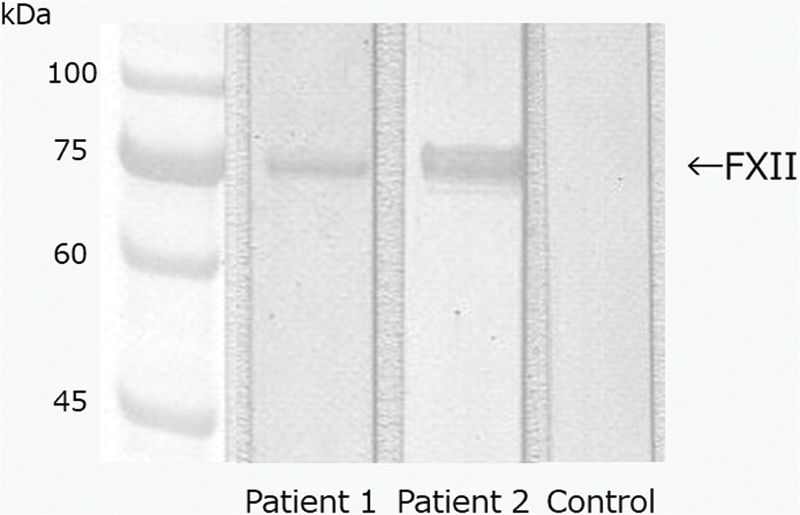
Binding of ASQ41-bound autoantibodies purified from patient plasma to FXII. FXII was subjected to SDS-PAGE under reducing conditions. FXII (8 µL of 20 µg/mL solution) was applied to each lane. Subsequent transfer to PVDF membranes was performed for 20 minutes at 0.1 amps. Membranes were then blocked for 2 hours with 1% BSA in TBS, pH 7.3. After incubation with the eluate (1:5) from the ASQ41-affinity column that contained autoantibodies to ASQ41 that were purified from anti-ASQ41-positive patient plasma or normal control plasma for 2 hours, we performed three washes in 0.05% Tween 20/TBS. The membranes were then exposed to horseradish peroxidase-conjugated polyclonal antibodies to human IgM for 1 hour, followed by washing as described above. The immunoreactive bands were developed using 3,3′,5,5′-tetramethylbenzidine. BSA, bovine serum albumin; FXII, factor XII; IgM, immunoglobulin M; PVDF, polyvinylidene difluoride; SDS-PAGE, sodium dodecyl sulfatepolyacrylamide gel electrophoresis; TBS, Tris-buffered saline.

As shown in Fig. 5 , the autoantibodies to ASQ41 purified from patient 1 and 2 recognized the whole FXII molecule under reducing conditions.

### Binding of ASQ41-Bound Autoantibodies Purified from Patient Plasma to Recombinant Mouse EGF, Human EGF, and HB-EGF

**Fig. 6.**
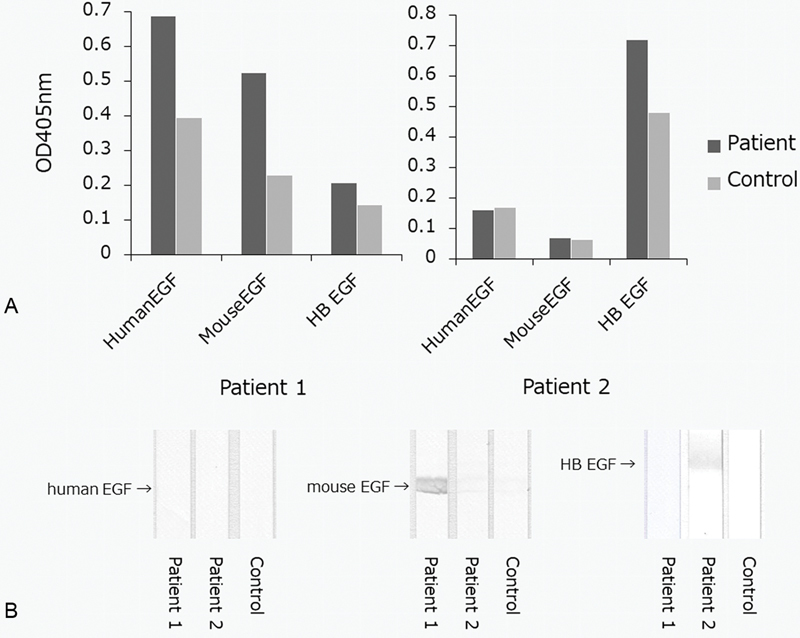
A representative experiment showing binding of ASQ41-bound autoantibodies purified from patient plasma to recombinant mouse EGF, human EGF, and HB-EGF. ( **A** ) ELISA was performed using amino plates coated with 10 µM recombinant mouse EGF, human EGF, or HB-EGF. The eluate (1:10) from the ASQ41-affinity column that contained autoantibodies to ASQ41 that were purified from anti-ASQ41-positive patient plasma or normal control plasma was applied, followed by an alkaline-phosphatase-conjugated secondary antibody. The absorption at 405 nm was then measured. ( **B** ) SDS-PAGE was performed using a polyacrylamide gradient gel (5–20%). Recombinant human EGF, mouse EGF, or human HB-EGF (30 μM solution) under reducing conditions was applied to each lane. Subsequent transfer to a PVDF membrane was performed for 20 minutes at 0.1 amps. We used 1% BSA in TBS (pH 7.3) to block the membrane for 1 hour. Incubation was performed with the eluate (1:10) from the ASQ41-affinity column that contained autoantibodies to ASQ41 that were purified from anti-ASQ41-positive patient plasma or normal control plasma for 2 hours, followed by three washes in 0.05% Tween 20/TBS. The membranes were exposed to horseradish-peroxidase-conjugated polyclonal antibodies against human IgM for 1 h, followed by washing as described above. The immunoreactive bands were developed using 3,3′,5,5′-tetramethylbenzidine. BSA, bovine serum albumin; EGF, epidermal growth factor; ELISA, enzyme-linked immunosorbent assay; HB, heparin binding; IgM, immunoglobulin M; PVDF, polyvinylidene difluoride; SDS-PAGE, sodium dodecyl sulfatepolyacrylamide gel electrophoresis; TBS, Tris-buffered saline.

As shown in Fig. 6A , the ODs of recombinant human EGF and mouse EGF in patient 1 were greater than those in normal control. The OD of recombinant human HB-EGF in patient 2 was greater than that in normal control.

As shown in Fig. 6B , the autoantibodies to ASQ41 purified from patient 1 recognized recombinant mouse EGF. The autoantibodies to ASQ41 purified from patient 2 recognized recombinant human HB-EGF.

### Polyclonal Antibodies against EGF Recognize FXII

**Fig. 7.**
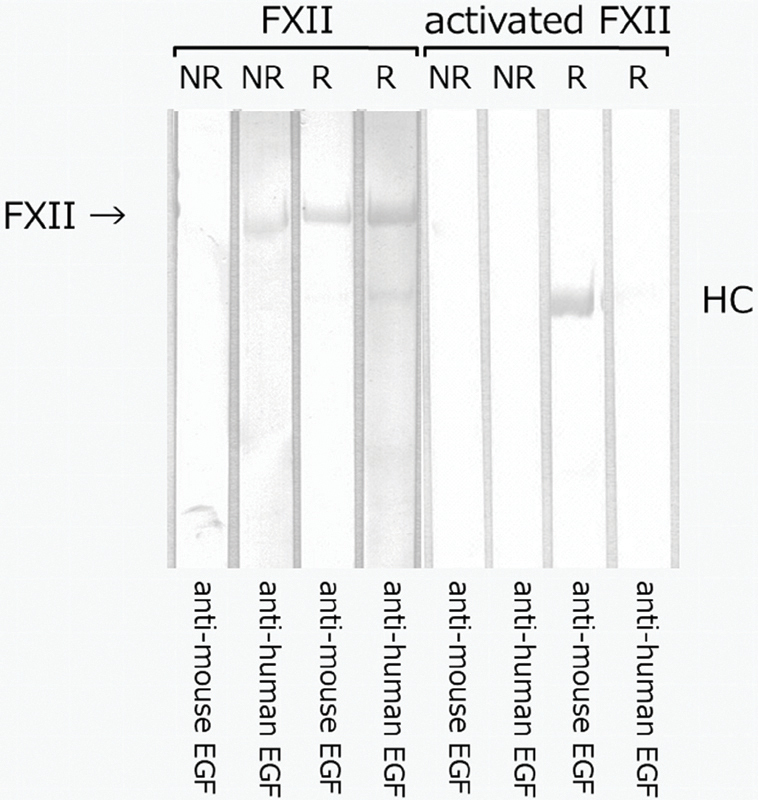
A representative experiment showing binding of polyclonal antibodies to EGF through FXII. FXII or activated FXII was subjected to SDS-PAGE under nonreducing (NR) or reducing (R) conditions. In reduced forms, activated FXII was divided into heavy chain and light chain. FXII or activated FXII (8 µL of 100 µg/mL solution) was applied to each lane. Subsequent transfer to PVDF membranes was performed for 20 minutes at 0.1 amps. Membranes were then blocked for 2 hours with 1% BSA in TBS, pH 7.3. After incubation with polyclonal antibodies to recombinant human EGF (antihuman EGF; 0.2 µg/mL) or mouse EGF (antimouse EGF; 0.2 µg/mL) for 2 hours, we performed three washes in 0.05% Tween 20/TBS. The membranes were then exposed to horseradish peroxidase-conjugated polyclonal antibodies to goat IgG for 1 hour, followed by washing as described above. The immunoreactive bands were developed using 3,3′,5,5′-tetramethylbenzidine. BSA, bovine serum albumin; EGF, epidermal growth factor; FXII, factor XII; HC: heavy chain; IgG, immunoglobulin G; PVDF, polyvinylidene difluoride; SDS-PAGE, sodium dodecyl sulfatepolyacrylamide gel electrophoresis; TBS, Tris-buffered saline.

As shown in Fig. 7 , antimouse EGF recognized the whole FXII molecule under reducing conditions and the heavy chain of activated FXII under reducing conditions. Antihuman EGF recognized the whole FXII molecule under both nonreducing and reducing conditions.

## Discussion

We previously reported that anti-FXII antibodies in the plasma of patients with recurrent pregnancy loss recognized the heavy chain of FXII but not the light chain. 11 In the present study, we revealed that autoantibodies in plasma of patients with recurrent pregnancy loss recognized the ASQ41 peptide that span EGF2 in the heavy chain of FXII. Anti-ASQ41 species were detected in 23 (23.0%) of 100 patients with recurrent pregnancy loss. Furthermore, among 23 anti-ASQ41-positive patients, plasma from 13 patients (56.5%) recognized a 22-residue segment in ASQ41, termed EGH22, which is equivalent to the C-terminal half of EGF2. We speculate that all plasma from 23 patients did not recognize EGH22 or ASQ19, because cleavage of ASQ 41 resulted in subtle changes in antigenicity. EGF2 or ASQ41 has complex three-dimensional structure with disulfide bonds. Therefore, antigenicity may be changed by cleavage of ASQ41. It may also be because the epitope is located on the boundary between ASQ19 and EGH22.

It is known that FXII is a coagulation factor, but several studies have suggested that FXII also functions as a growth factor and mediates cell signaling, which leads to proliferation and stimulates angiogenesis. 20 21 22 FXII has been shown to enhance the proliferation of human hepatoma cells, 20 to activate signal transduction pathways, and to exert mitogenic effects on several EGF-sensitive cell types. 21 Additionally, it has been reported that FXII stimulates Akt phosphorylation and extracellular signal-related kinase 1/2 (ERK1/2) through the EGF receptor, specific integrins, and urokinase plasminogen activator receptor (uPAR), which leads to proliferation, growth, and angiogenesis of endothelial cells. 22

In this study, we suggested that anti-FXII antibodies in patients with recurrent pregnancy loss recognized the EGF2 domain in FXII. Recently, Velayuthaprabhu et al reported that, in a murine model, anti-FXII antibody infusion increased placental apoptosis and the number of shrunken cells in the decidua and decreased the number of mitotic cells and invasiveness of trophoblast giant cells. 16 Anti-EGF2 antibodies in patients with recurrent pregnancy loss may inhibit the growth factor functions of FXII and cause placental dysfunction.

Proteins in the EGF family play an important role in pregnancy. Accumulating evidence suggests that the survival and invasive capacities of human trophoblasts are associated with intercellular downstream signaling related to the EGF family. EGF protects human term cytotrophoblast cells against apoptosis during culture in vitro, 23 24 which indicates that EGF can act as a survival factor. Studies of first trimester cytotrophoblast cells cultured in vitro suggest that EGF, HB-EGF, and transforming growth factor alpha (TGFA) stimulate motility and invasiveness of trophoblast cells. 25 26 HB-EGF can also protect first trimester cytotrophoblast cells against apoptosis when they are exposed to low oxygen levels 27 or oxidative stress due to hypoxia or reoxygenation injury. 28

In this study, we found that autoantibodies to ASQ41 in patients with recurrent pregnancy loss recognized EGF family proteins such as EGF and HB-EGF. We generated polyclonal antibodies to ASQ41 by immunizing rabbits with the ASQ41 peptide. Interestingly, these polyclonal antibodies did not recognize human EGF, mouse EGF, or human HB-EGF (data not shown). Moreover, polyclonal antibodies against human and mouse EGF did not recognize ASQ41 (data not shown). EGF family proteins have complex three-dimensional structure with disulfide bonds. Antigenicity may be changed between the liquid and solid phase, between ELISA and immunoblotting, and also between reducing and nonreducing conditions.

It is known that thrombophilia is associated with early and late pregnancy complications, and numerous studies have been performed to investigate the effects of thrombophilia on pregnancy. This study focused on FXII as a growth factor and indicated that autoantibodies in patients with recurrent pregnancy loss may not only cause thrombophilia-associated complications but may also disrupt the EGF system.

In this study, we did not screen for anti-beta-2 glycoprotein I antibodies. No patient was diagnosed as antiphospholipid syndrome (APS) because we did not do the second blood tests to confirm positive results. Further studies about association between autoantibodies to EGF family and APS are necessary.

The incidences of FXII deficiency and protein S (PS) deficiency in the Japanese population are relatively high, and both are undeniable risk factors for spontaneous abortion. 29 30 Recently, Ebina et al reported that low levels of PS and FXII were related to adverse pregnancy outcomes, such as pre-eclampsia, pregnancy-induced hypertension, and premature delivery. 30 PS plays an important role in many biological processes, including coagulation, angiogenesis, vasculogenesis, apoptosis, and cancer progression. 31 It was reported that PS surrounds damaged placental trophoblast cells in both early and late pregnancy. This indicates that PS can restore or protect damaged villi and that it may exert physiological effects on the placenta. 32

Interestingly, our recent study revealed that autoantibodies to PS (anti-PS) were detected in 20 (20.0%) of 100 patients with recurrent pregnancy loss and that anti-PS recognized EGF-like domains of PS. 33 Furthermore, among 20 anti-PS-positive patients, 14 patients (70.0%) recognized mouse EGF. 33 Anti-PS in patients with recurrent pregnancy loss may therefore inhibit functions of PS as a growth factor.

In conclusion, autoantibodies in patients with recurrent pregnancy loss recognize EGF-like domains in various proteins and members of the EGF family. Autoantibody-associated disruption of the EGF system might be a cause of recurrent pregnancy loss. This is a novel hypothesis for the pathogenesis of autoantibodies in patients with recurrent pregnancy loss.

## References

[1] Schved J F Gris J C Neveu S Dupaigne D Mares P Factor XII congenital deficiency and early spontaneous abortion Fertil Steril 1989 52 02 335 336 2753183 10.1016/s0015-0282(16)60866-x

[2] Braulke I Pruggmayer M Melloh P Hinney B Köstering H Günther E Factor XII (Hageman) deficiency in women with habitual abortion: new subpopulation of recurrent aborters? Fertil Steril 1993 59 01 98 101 8419231 10.1016/s0015-0282(16)55622-2

[3] Gris J C Ripart-Neveu S Maugard C Respective evaluation of the prevalence of haemostasis abnormalities in unexplained primary early recurrent miscarriages. The Nimes Obstetricians and Haematologists (NOHA) Study Thromb Haemost 1997 77 06 1096 1103 9241739

[4] Gallimore M J Jones D W Winter M Factor XII determinations in the presence and absence of phospholipid antibodies Thromb Haemost 1998 79 01 87 90 9459329

[5] Jones D W Gallimore M J Harris S L Winter M Antibodies to factor XII associated with lupus anticoagulant Thromb Haemost 1999 81 03 387 390 10102466

[6] Matsuura T Kobayashi T Asahina T Kanayama N Terao T Is factor XII deficiency related to recurrent miscarriage? Semin Thromb Hemost 2001 27 02 115 120 11372764 10.1055/s-2001-14069

[7] Pauer H U Renné T Hemmerlein B Targeted deletion of murine coagulation factor XII gene-a model for contact phase activation in vivo Thromb Haemost 2004 92 03 503 508 15351846 10.1160/TH04-04-0250

[8] Iwaki T Castellino F J Plasma levels of bradykinin are suppressed in factor XII-deficient mice Thromb Haemost 2006 95 06 1003 1010 16732380 10.1160/TH06-03-0128

[9] Jones D W Gallimore M J MacKie I J Harris S L Winter M Reduced factor XII levels in patients with the antiphospholipid syndrome are associated with antibodies to factor XII Br J Haematol 2000 110 03 721 726 10997986 10.1046/j.1365-2141.2000.02251.x

[10] Jones D W MacKie I J Gallimore M J Winter M Antibodies to factor XII and recurrent fetal loss in patients with the anti-phospholipid syndrome Br J Haematol 2001 113 02 550 552 11380430 10.1046/j.1365-2141.2001.02776.x

[11] Inomo A Sugi T Fujita Y Matsubayashi H Izumi S Mikami M The antigenic binding sites of autoantibodies to factor XII in patients with recurrent pregnancy losses Thromb Haemost 2008 99 02 316 323 18278180 10.1160/TH07-07-0447

[12] Bradford H N Pixley R A Colman R W Human factor XII binding to the glycoprotein Ib-IX-V complex inhibits thrombin-induced platelet aggregation J Biol Chem 2000 275 30 22756 22763 10801853 10.1074/jbc.M002591200

[13] Clarke B J Côté H CF Cool D E Mapping of a putative surface-binding site of human coagulation factor XII J Biol Chem 1989 264 19 11497 11502 2472397

[14] Pixley R A Stumpo L G Birkmeyer K Silver L Colman R W A monoclonal antibody recognizing an icosapeptide sequence in the heavy chain of human factor XII inhibits surface-catalyzed activation J Biol Chem 1987 262 21 10140 10145 2440859

[15] Sato Y Sugi T Sakai R Autoantibodies to factor XII and kininogen-dependent antiphosphatidylethanolamine antibodies in patients with recurrent pregnancy loss augment platelet aggregation Am J Reprod Immunol 2015 74 03 279 289 26011374 10.1111/aji.12402

[16] Velayuthaprabhu S Matsubayashi H Sugi T A unique preliminary study on placental apoptosis in mice with passive immunization of anti-phosphatidylethanolamine antibodies and anti-factor XII antibodies Am J Reprod Immunol 2011 66 05 373 384 21623987 10.1111/j.1600-0897.2011.01008.x

[17] Sugi T McIntyre J A Autoantibodies to phosphatidylethanolamine (PE) recognize a kininogen-PE complex Blood 1995 86 08 3083 3089 7579402

[18] Sugi T Mclntyre J A Phosphatidylethanolamine induces specific conformational changes in the kininogens recognizable by antiphosphatidylethanolamine antibodies Thromb Haemost 1996 76 03 354 360 8883270

[19] Katsunuma J Sugi T Inomo A Matsubayashi H Izumi S-I Makino T Kininogen domain 3 contains regions recognized by antiphosphatidylethanolamine antibodies J Thromb Haemost 2003 1 01 132 138 12871550 10.1046/j.1538-7836.2003.00022.x

[20] Schmeidler-Sapiro K T Ratnoff O D Gordon E M Mitogenic effects of coagulation factor XII and factor XIIa on HepG2 cells Proc Natl Acad Sci U S A 1991 88 10 4382 4385 1852005 10.1073/pnas.88.10.4382 PMC51663

[21] Gordon E M Venkatesan N Salazar R Factor XII-induced mitogenesis is mediated via a distinct signal transduction pathway that activates a mitogen-activated protein kinase Proc Natl Acad Sci U S A 1996 93 05 2174 2179 8700904 10.1073/pnas.93.5.2174 PMC39930

[22] LaRusch G A Mahdi F Shariat-Madar Z Factor XII stimulates ERK1/2 and Akt through uPAR, integrins, and the EGFR to initiate angiogenesis Blood 2010 115 24 5111 5120 20228268 10.1182/blood-2009-08-236430 PMC2890145

[23] Payne S G Brindley D N Guilbert L J Epidermal growth factor inhibits ceramide-induced apoptosis and lowers ceramide levels in primary placental trophoblasts J Cell Physiol 1999 180 02 263 270 10395296 10.1002/(SICI)1097-4652(199908)180:2<263::AID-JCP14>3.0.CO;2-H

[24] Smith S Francis R Guilbert L Baker P N Growth factor rescue of cytokine mediated trophoblast apoptosis Placenta 2002 23 04 322 330 11969343 10.1053/plac.2001.0783

[25] Bass K E Morrish D Roth I Human cytotrophoblast invasion is up-regulated by epidermal growth factor: evidence that paracrine factors modify this process Dev Biol 1994 164 02 550 561 8045351 10.1006/dbio.1994.1223

[26] Leach R E Kilburn B Wang J Liu Z Romero R Armant D R Heparin-binding EGF-like growth factor regulates human extravillous cytotrophoblast development during conversion to the invasive phenotype Dev Biol 2004 266 02 223 237 14738873 10.1016/j.ydbio.2003.09.026

[27] Armant D R Kilburn B A Petkova A Human trophoblast survival at low oxygen concentrations requires metalloproteinase-mediated shedding of heparin-binding EGF-like growth factor Development 2006 133 04 751 759 16407398 10.1242/dev.02237 PMC1679956

[28] Leach R E Kilburn B A Petkova A Romero R Armant D R Diminished survival of human cytotrophoblast cells exposed to hypoxia/reoxygenation injury and associated reduction of heparin-binding epidermal growth factor-like growth factor Am J Obstet Gynecol 2008 198 04 4710 4.71E9, discussion 471.e7–471.e8 10.1016/j.ajog.2008.01.009 PMC2390925 18395045

[29] Sakata T Okamoto A Mannami T Tomoike H Miyata T Prevalence of protein S deficiency in the Japanese general population: the Suita Study J Thromb Haemost 2004 2 06 1012 1013 15140145 10.1111/j.1538-7836.2004.00742.x

[30] Ebina Y Ieko M Naito S Low levels of plasma protein S, protein C and coagulation factor XII during early pregnancy and adverse pregnancy outcome Thromb Haemost 2015 114 01 65 69 25879167 10.1160/TH14-11-0928

[31] Suleiman L Négrier C Boukerche H Protein S: a multifunctional anticoagulant vitamin K-dependent protein at the crossroads of coagulation, inflammation, angiogenesis, and cancer Crit Rev Oncol Hematol 2013 88 03 637 654 23958677 10.1016/j.critrevonc.2013.07.004

[32] Matsumoto M Tachibana D Nobeyama H Protein S deposition at placenta: a possible role of protein S other than anticoagulation Blood Coagul Fibrinolysis 2008 19 07 653 656 18832905 10.1097/MBC.0b013e3283001d1f

[33] Sato Y Sugi T Sakai R Antigenic binding sites of anti-protein S autoantibodies in patients with recurrent pregnancy loss Res Pract Thromb Haemost 2018 2 02 357 365 30046739 10.1002/rth2.12081 PMC6055483

